# Evaluation of Hemodynamic Parameters as Predictors of Glaucoma Progression

**DOI:** 10.1155/2011/164320

**Published:** 2011-04-10

**Authors:** Ingrida Janulevičiene, Rita Ehrlich, Brent Siesky, Irena Nedzelskienė, Alon Harris

**Affiliations:** ^1^Eye Clinic, Kaunas University of Medicine, Eiveniu Street 2, 50009 Kaunas, Lithuania; ^2^Department of Ophthalmology, Glaucoma Research and Diagnostic Center, Indiana University School of Medicine, 702 Rotary Circle, Room 137, Indianapolis, IN 46202, USA; ^3^Biostatistician, Faculty of Odontology, Kaunas University of Medicine, 50106 Kaunas, Lithuania

## Abstract

*Purpose*. To evaluate hemodynamic parameters as possible predictors for glaucoma progression. *Methods*. An 18-month randomized double-masked cohort study including 30 open-angle glaucoma patients receiving fixed-combination treatment with Dorzolamide/Timolol (DTFC) or Latanoprost/Timolol (LTFC) (*n* = 15 per group) was performed. Intraocular pressure (IOP), arterial blood pressure (BP), ocular and diastolic perfusion pressures (OPP, DPP), color Doppler imaging, pulsatile ocular blood flow analysis, scanning laser polarimetry, and Humphrey visual field evaluations were included. *Results*. Both treatments showed statistically similar IOP reduction. Six patients in DTFC and 7 in LTFC group met glaucoma progression criteria. DTFC group had higher OPP, DPP, and lower vascular resistivity indices as compared to the LTFC. Progressing patients had higher nerve fiber index, lower systolic BP, OPP, DPP, higher ophthalmic and central retinal artery vascular resistance, and lower pulse volume (*P* < .05; *t*-test). *Conclusions*. Structural changes consistent with glaucoma progression correlate with non-IOP-dependent risk factors.

## 1. Introduction

The recent series of large, multicenter, randomized clinical trials examining glaucoma treatment provide some information regarding current management goals for maintaining a target intraocular pressure (IOP). However, in many cases, glaucoma progression occurs despite maintaining target IOP. For instance, in the Collaborative Normal-Tension Glaucoma (CNTG) study, 12 to 18% of glaucoma patients progressed despite a 30% IOP reduction [1]; in the Early Manifest Glaucoma Trial (EMGT), 45% progressed despite an average IOP reduction of 25% at 6-year followup [2]. Leske et al. [3] further reported that 67% of patients progressed over 11 years of followup despite IOP reduction. 

Non-IOP factors have also been identified as contributing to open-angle glaucoma (OAG) progression, including lower ocular perfusion pressure (OPP), reduced ocular blood flow, cardiovascular disease, and low systolic blood pressure. Impaired optic nerve blood flow is considered a potential causative factor in the development of glaucoma optic neuropathy [4, 5]. However, it remains unknown whether manipulation of perfusion pressure, blood pressure, and ocular blood flow will prevent glaucoma progression.

The European Glaucoma Guidelines of 2008 [6] set the preservation of visual function as the primary goal of glaucoma therapy. In cellular terms, this can be interpreted as prevention of retinal ganglion cell death. However, the exact factors contributing to retinal ganglion cell death remain speculative [7]. Although changes in ocular blood flow might be the consequence of IOP variations, they can also be a primary physiological event [8]. As IOP therapies may influence ocular perfusion [9], it is vital to investigate glaucoma therapies for vascular interactions in addition to IOP reduction. One possible therapy is dorzolamide hydrochloride, a potent vasoactive glaucoma topical treatment that many studies have shown to increase various measures of ocular blood flow [10–16]. Although not all studies are in full agreement [17, 18], a recent meta-analysis of published studies found carbonic anhydrase inhibitors, such as dorzolamide, to be consistently effective at increasing the ocular circulation [19].

Much less research has been conducted to investigate the effects of a combination treatment on improving ocular circulation and reducing IOP, especially in relation to glaucoma progression. To our knowledge, there are no long-term prospective double-blind studies that investigated the IOP lowering effects of fixed combinations and the correlation between ocular hemodynamic and both functional and structural changes in glaucoma patients. This study investigates the fixed combinations of dorzolamide/timolol (DTFC) and latanoprost/timolol (LTFC) on IOP lowering and glaucoma progression while examining if baseline ocular blood flow parameters are predictive of glaucomatous progression as determined by visual field and/or structural changes.

## 2. Materials and Methods

Thirty OAG patients were followed for 18 months in an observational cohort study. All subjects read and signed an informed consent, and the study was approved by the Kaunas University of Medicine institutional review board. Inclusion criteria: OAG patients with characteristic glaucomatous visual field loss, optic nerve head damage, and IOP not adequately controlled with timolol maleate (BID). Exclusion criteria: mean deviation worse than or equal to −12 dB in Humphrey Visual Fields (HVFA) central 24-2 SITA Standard, cup to disc ratio equal or greater than 0.9, history of eye disease other than refractive error, orbital or ocular trauma, history of renal or hepatic disease, asthma or respiratory disease, allergy to either of the study medications, and pregnant or nursing women. After timolol baseline examination, patients were randomly assigned to double masked fixed combination treatment: LTFC or DTFC. Examinations were carried out in both eyes and the study eye was chosen randomly. All study visits were scheduled at the same time of day ±1 hour in order to avoid diurnal fluctuations in IOP and arterial BP. 

Examinations were carried out at baseline, 1, 6, 12, and 18 months of treatment, including full ophthalmic examination, visual acuity, Goldmann IOP, central corneal thickness (CCT) (OcuScan PXP Alcon Labs. Inc), Humphrey visual field examination (24-2 SITA Standard), and scanning laser polarimetry (GDx VCC Laser Diagnostic Technologies Inc., San Diego, CA). In the scanning laser polarimetry scan printout each color represents a different probability of the parameter being outside normal limits, with red having the highest probability (*P* < .005), followed by yellow (*P* < .01), light blue (*P* < .02), and dark blue (*P* < .05); green (*P* < .05) refers to normal limits. 

All patients had 5 or more visual fields and scanning laser polarimetry scans for analysis. Glaucoma progression was identified by (1) standard automated perimetry (SAP) as a statistically significant decrease from baseline examination in the pattern deviation values. Deepening of an existing scotoma was considered if two points in an existing scotoma declined by ≥10 dB. Expansion of an existing scotoma was considered if two contiguous points adjacent to an existing scotoma declined by ≥10 dB. A new scotoma was diagnosed if an alteration meeting the criteria for glaucomatous visual field defect occured in previously normal visual field location. Three or more locations with *P* < .01 constituted a change of threshold sensitivity. (2) Progressive optic disc change is determined by optic disc assessment by ophthalmoscopy and scanning laser polarimetry. Advanced Serial Analysis detected repeatable change on two consecutive scans compared with baseline images using thickness map, and deviation map, deviation from reference map, temporal-superior-nasal-inferior-temporal (TSNIT) graph or a significant change in slope of the summary parameter chart. Each slope represented the change in RNFL thickness per year, assuming a linear trend across the followup period [3, 20–22]. 

Ocular blood flow was evaluated with pulsatile ocular blood flow analyser POBF (Paradigm medical industries. Inc.) and Color Doppler imaging (CDI) (Accuvix XQ. Medison Co., LTD. Seoul, Republic of Korea). Blood flow velocities were measured in the ophthalmic (OA), central retinal (CRA), and short posterior ciliary arteries (SPCA), with a 7.5 MHz linear probe calculating peak systolic velocity (PSV), end-diastolic velocity (EDV), and resistive index (RI) in each vessel. Vascular RI was originally described by Pourcelot and is calculated as RI = (PSV − EDV)/PSV [23–26].

All patients' data were collected in the Eye Clinic of Kaunas Medical University (Lithuania). CDI readings were performed by a Reading Center: the Glaucoma Research and Diagnostic Laboratories in the Department of Ophthalmology, Indiana University School of Medicine (USA).

## 3. Statistical Analysis

CDI presents 12 different parameters with a coefficient of variation ranging from 1.7% to 18%, and the majority of parameters present with a coefficient of variation under 10%. The coefficient of variation for total RNFL thickness is 5%. With a sample size of 15 in each group, we have at least 90% power to detect a change as small as 8.5% with alpha level 0.05 in retrobulbar velocities and 4.2% in RNFL thickness. The coefficient of variation for POBF is 15% [24]. In this analysis, we determined our sample size must be greater than 29.17 subjects to detect changes smaller than 9% in blood flow parameters. Changes in visual fields over time were analyzed using Humphrey's STATPAC software as described in Materials and Methods.

Descriptive statistics were obtained for the resulting measurements. In the event that significance was achieved by repeated ANOVA measurements, we applied the Fisher's and Bonferroni models. Changes in individual parameters were examined by paired Student's *t*-test. *P* values of *P* < .05 were considered statistically significant. To test the hypothesis that the mean difference between two measurements is zero, Wilcoxon signed-ranks test was used. Changes in OBF and glaucomatous optic neuropathy parameters (functional and structural changes) were analyzed by Pearson's correlation analysis. Multivariate regression models were used to evaluate potential risk factors for glaucoma progression: age, IOP, systolic BP, diastolic BP, OPP, DPP, pulse volume, and RI of retrobulbar vessels. Receiver Operating Characteristic (ROC) curves for progressing glaucoma patients were performed to analyze the discriminating ability of possible vascular risk factors.

## 4. Results

We examined 30 OAG patients (15 patients in each study group) with a mean age of 58.13 (SD 8.6), including 5 males and 25 females. There were no statistically significant differences between baseline parameters of either treatment group.

Both DTFC and LTFC had similar IOP lowering effect over 18 months of observation (*P* = .653; *t*-test). Baseline systolic and diastolic BP were comparable between DTFC and LTFC groups (*P* = 0.101 and *P* = 0.07, resp., *t*-test). DTFC showed statistically significantly higher OPP, SPP, and DPP at 1, 6, and 18 months visits (Table 1). 

CDI baseline retrobulbar blood flow parameters were similar between the two groups (*P* > .05; *t*-test), except for a statistically significantly higher OA-PSV and CRA-EDV in the LTFC group (Table 2). Both combination treatment regimes increased retrobulbar blood flow velocities compared to baseline, though significant changes from baseline at the OA-PSV (*P* = .003), OA-EDV (*P* = .001), and CRA-PSV (*P* = .001) were only seen in the DTFC group at 1- and 12-month followup. Vascular RI were decreased in the DTFC group, showing statistically significantly lower resistivity compared to the LTFC group in the CRA and SPCA during 12- and 18-month visits (Table 2). CRA-PSV correlated with OA-PSV (*r* = 0.505; *P* = .004) and OA-EDV (*r* = 0.450; *P* = .013), and SPCA-EDV correlated with DBP (*r* = 0.454; *P* = .012), DPP (*r* = 0.449; *P* = .013), and OA-RI (*r* = −0.432; *P* = .017). 

Average IOP, pulse amplitude, and POBF were not statistically different between treatment arms (Table 3). Pulse volume increases in the DTFC group and differences at the 12- and 18-month visits when compared to the LTFC group were significant (*P* = .025 and *P* = .054, resp.). 

Glaucoma progression was identified in 13 eyes (21.7%): 4 (6.7%) exhibiting structural changes, 1 (1.7%) with perimetric changes, and 8 (13.3%) showing both perimetric and structural changes. There were no statistically significant differences in IOP between progressing and stable glaucoma patients at the final visit (Table 4). Progressing glaucoma patients had higher OA RI, lower SPCA-EDV (*P* < .05; *t*-test), and decreased pulse volume by 2.68 (SD 0.61)  *μ*L (*P* = .0001; *t*-test) as compared to stable glaucoma patients at the 18-month visit. Progressing glaucoma cases had significantly lower SBP, OPP, and DPP (Table 4). 

Changes in TSNIT correlated with SBP (*r* = 0.614; *P* = .025) in progressing glaucoma patients. The odds of higher NFI at the final 18-month visit was 13.82 times greater (95% CI 1.32–143.76) in patients with baseline CRA RI ≥ 0.67 (*P* = .028) and older age patients (95% CI 0.90–0.99) (*P* = .021).

**Figure 1:**
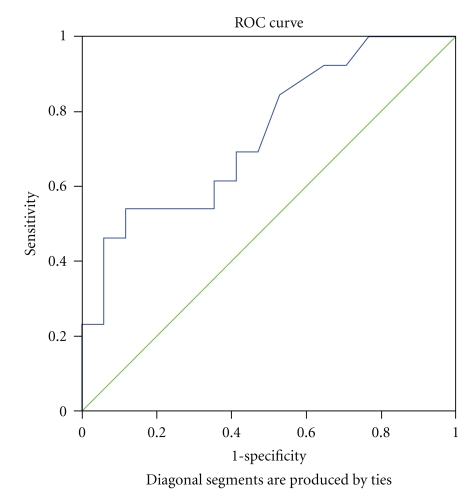
ROC curve—DPP at 18-month visit in progressing glaucoma patients. ROC: Receiver operating characteristic.

**Figure 2:**
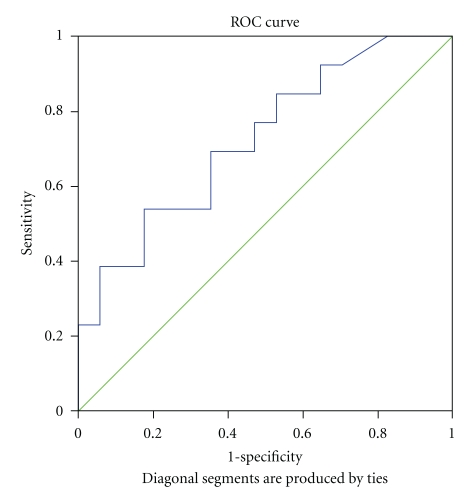
ROC curve—OPP at 18-month visit in progressing glaucoma patients. ROC: Receiver operating characteristic.

The area under the Receiver Operating Characteristic (ROC) curve in progressing glaucoma patients with DPP < 62 mmHg was 0.74 (95% CI lower bound 0.56; upper bound 0.919; *P* = .027) (Figure 1); the sensitivity and specificity were 0.385 and 0.941, respectively. Progressing glaucoma patients with OPP < 52 mmHg had an area under the ROC curve of 0.72 (95% CI lower bound 0.54; upper bound 0.907; *P* = .038) (Figure 2); the sensitivity and specificity were 0.385 and 0.941, respectively. In our analysis, we found power 0.88 with type I error of 0.05 and, although sensitivity was low at cut off, the specificity was high.

## 5. Discussion

This observational cohort study showed that despite the IOP lowering effect with different fixed combinations (DTFC and LTFC), 13 eyes (21.7%) were considered as progressing glaucoma during 18 months of observation. Among patients with progressing glaucoma, 6 were with DTFC and 7 with LTFC treatment and showed no statistically significant hypotensive effect between the two fixed combinations. Evidence shows that despite a wide range of glaucoma therapy options to reduce IOP, it is still difficult in some cases to control slowly progressing optic neuropathy. During our 18-month observation, no cases of intolerance were found and all patients completed the study.

Previously, Siesky et al. [27] reported that DTFC increased ocular blood flow in OAG patients while attaining a similar IOP reduction compared to a treatment of latanoprost plus timolol. Visual function, as expected, was not different in this short-term comparison. Evidence of decreased optic nerve blood flow correlating with visual field damage has been reported in glaucoma patients [28–33]. In our study, we report differences in OPP and DPP between DTFC and LTFC; however, no significant differences were observed between LTFC and DTFC in terms of glaucoma progression during the 18-month followup. 

Previous studies examining ocular blood flow and glaucoma progression reported structural abnormalities [34] preceding visual field damage. Hafez et al. [35] also concluded that rim perfusion might be reduced before manifestation of visual field defects. Several studies have shown abnormal retrobulbar vasculature in eyes with Glaucomatous Optic Neuropathy (GON) [36–40]. Satilmis et al. [41] showed that progression rate of glaucomatous visual field damage correlates with retrobulbar hemodynamic variables. Zeitz et al. [42] further showed that progressive glaucoma is associated with decreased blood flow velocities in the small retrobulbar vessels supplying the optic nerve head. We found increased blood flow velocities with combination treatment as compared to timolol baseline. DTFC arm had statistically significantly lower baseline OA-PSV and CRA-EDV as compared to LTFC baseline. After 1, 6, 12, and 18 months of combination treatment, the velocities in retrobulbar vessels increased as compared to baseline, but differences in velocities between two treatment arms were not statistically significant. In our study, SPCA-EDV was lower in progressing glaucoma patients as compared to stable glaucoma patients. We found statistically significant differences in RIs between the two treatment cohorts. DTFC showed statistically significant decrease in CRA and SPCA RIs at 12- and 18-month visits as compared to LTFC. Nielsen and Nyborg [43] found that PG F2*α* induces constriction in isolated bovine aqueous veins. Remky et al. [44] reported that reduction in retinal vessel diameters may account for an increase in retinal vascular resistance. An increase in vascular resistance might be related to vasoconstriction or vasospasm, vasosclerosis, reduction of the vessel diameters, or rheological factors leading to decreased volumetric flow. In our study, POBF that measures pulse volume was significantly higher in DTFC at 12 and 18-month visits compared to LTFC. Progressing glaucoma patients had 2.675 (SD 0.61)  *μ*L lower pulse volume when compared to stable glaucoma cases (*P* = .0001). Our results indicate DTFC indeed increases markers of ocular blood flow and perfusion compared to LTFC but with no difference in possible markers of glaucoma progression during the followup period. Longer duration studies may be required to differentiate any possible (or lack thereof) ocular blood flow benefits. 

The Beaver Dam study reported a positive correlation between systolic BP and IOP [45]. The Los Angeles Latino Eye Study [46] showed high systolic BP, low diastolic BP, and low OPP as risk factors for glaucoma progression. Data from EMGT [3] pointed to low systolic BP as a long-term predictor for glaucoma progression. Further, data from Thessaloniki Eye study [47] suggested BP status as an important independent factor initiating optic disc changes and/or as a contributing factor to glaucoma damage. In our study, we found no fluctuations or rise in IOP, but OPP and DPP at 1, 6, and 18-month visits were statistically significantly higher in the DTFC group. The LTFC group had lower SBP at 1, 6, and 18-month visits and diastolic BP at 1 and 6 month visits (*P* < .05; *t*-test). Progressing patients had statistically significantly lower systolic BP, OPP, and DPP when compared to stable glaucoma cases. Calculating the magnitude of changes in OPP and DPP parameters compared to baseline values, we found them to be decreasing in 69.2% of progressing glaucoma cases. Our calculated sensitivity of decreased DPP was 0.7 and specificity 0.8. 

BP and ocular perfusion pressure tend to exhibit fluctuations during the day and night. Importantly, Choi et al. [48] reported that mean BP and OPP fluctuations were associated with reduced TSNIT and increased NFI. In our study, BP was measured at the same time of the day during all visits and statistically significant differences in BP and OPP parameters were seen at 1, 6, and 18 months but were not significant at 12 months between the two treatment groups. The LTFC group showed lower OPP and DPP and higher NFI as compared to DTFC at the 18 month visit (mean difference 7.80 (SD 3.69) (*P* = .046). Accordingly, progressing glaucoma patients showed lower OPP and DPP and higher NFI (mean difference 8.87 (SD 3.94)); *P* = .056). Yet, despite differences in the nonpressure-related parameters, we found no difference in the percent of progression between the treatment groups. In addition, we also found a strong positive correlation between TSNIT average and BP and OPP parameters at 18-month visit. Interestingly, low OPP and DPP in progressing glaucoma patients had low sensitivity but rather high specificity. In our analysis, statistically significant Area Under ROC Curve (AUC) values were reported at 0.74 and 0.72. While significant, these values should be further validated with a larger sample allowing for stratification into classified percentile ranges. The odds of higher NFI at the final 18-month visit was nearly 14 times greater in patients with higher than 0.67 baseline CRA RI (*P* = .028) and older age (*P* = .021).

Current glaucoma medications are targeted to decrease the IOP and are not targeted to treat other hemodynamic parameters. In our study, we found some differences in structural outcomes between the two combination treatment regimes and according differences in BP, OPP, CRA, and SPCA RIs. Our study is a preliminary study and the data presented needs to be interpreted with caution. Increased resistance to flow in small retrobulbar vessels supplying the optic nerve is probably related to glaucoma progression, although this requires confirmation in larger longitudinal studies.

Possible limitations of the current study include the difficulty in defining glaucoma progression and specific limitations in each imaging technology used to assess ocular blood flow. We have matched markers of possible glaucoma progression, which may indicate but not actually represent glaucomatous progression. While the parameters may be associated with progression, they are not necessarily good in predicting progression. A risk factor must be strongly associated with a disorder to be a worthwhile screening test, and it is not unusual for a strong risk factor to fail to be a good screening tool. Larger group studies with longer followup, standardization of measurement techniques for glaucoma progression, and ocular blood flow parameters are required to elicit a clear understanding of vascular risk factors in glaucoma progression.

##  Conflict of Interests

The authors have no proprietary interest in any aspect of the products or devices mentioned herein.

## Figures and Tables

**Table 1:** Comparison of characteristics of patients treated with DTFC and LTFC.

Characteristics	DTFC	LTFC	*P* value (*t*-test)
Age	56.93 (9.54)	59.33 (7.7)	.455
CCT (*μ*)	548.03 (39.86)	549.65 (41.71)	.914
C/D ratio	0.62 (0.14)	0.65 (0.15)	.576
SBP mmHg baseline	157.70 (14.90)	146.70 (20.22)	.101
1 month	152.73 (16.90)	136.00 (13.67)	.006*
6 months	161.80 (18.40)*	146.800 (15.40)*	.022*
12 months	148.500 (11.18)	144.200 (17.41)	.428
18 months	158.63 (14.24)	141.10 (15.21)	.003*
DBP mmHg baseline	92.13 (8.12)	86.80 (7.53)	.073
1 month	93.73 (15.41)	81.10 (7.04)	.009*
6 months	97.43 (12.19)*	86.87 (9.49)*	.013*
12 months	91.07 (8.47)	86.57 (9.10)	.172
18 months	88.80 (5.81)	83.83 (8.41)	.070
IOP mmHg baseline	22.10 (2.69)	20.57 (3.25)	.171
1 month	16.33 (2.11)	14.90 (2.69)	.116
6 months	16.17 (2.81)	14.70 (2.57)	.147
12 months	17.10 (2.42)	15.13 (3.42)	.080
18 months	16.17 (2.08)	15.70 (3.38)	.653
OPP mmHg baseline	53.8933 (5.61)	50.6100 (7.52)	.186
1 month	59.27 (9.70)*	51.47 (4.6)*	.011*
6 months	62.93 (8.98)*	56.33 (5.84)*	.024*
12 months	56.38 (6.19)	55.38 (6.92)	.683
18 months	57.56 (3.81)	52.18 (7.26)	.019*
SPP mmHg baseline	135.60 (7.40)	126.13 (10.51)	.008*
1 month	136.40 (12.1)	121.10 (7.5)	.003*
6 months	145.63 (19.6)	132.10 (8.4)	.020*
12 months	131.4 (8.25)	129.07 (10.24)	.498
18 months	142.46 (7.4)	125.40 (9.34)	.0001*
DPP mmHg baseline	70.03 (7.40)	66.23 (8.11)	.191
1 month	77.20 (15.12)*	66.73 (5.35)*	.021*
6 months	81.33 (12.19)*	71.67 (7.95)*	.016*
12 months	73.97 (8.41)	71.43 (8.90)	.430
18 months	72.97 (6.15)	66.03 (11.03)	.045*

*P < .05 statistically significant.

DTFC: dorzolamide/timolol fixed combination; LTFC: latanoprost/timolol fixed combination; CCT: central corneal thickness; C/D ratio: clinically determined cup-disc ratio; SBP: systolic blood pressure; DBP: diastolic blood pressure; IOP: intraocular pressure; OPP: ocular perfusion pressure; DPP: diastolic perfusion pressure.

**Table 2:** Color Doppler imaging parameters during 18 months of followup.

Characteristics		DTFC	LTFC	*P* value (*t*-test)
OA_PSV (cm/s)	Baseline	23.79 (8.837)	30.86 (9.30)	.042
	1 month	37.10 (12.33)	36.04 (7.83)	.781
	6 months	38.15 (16.24)	33.87 (8.27)	.371
	12 months	40.66 (15.51)	42.50 (14.01)	.736
	18 months	33.70 (10.05)	28.71 (6.93)	.125

OA_EDV (cm/s)	Baseline	4.82 (2.47)	7.03 (3.60)	.06
	1 month	8.22 (4.22)	8.78 (3.94)	.710
	6 months	8.87 (6.03)	7.66 (2.52)	.479
	12 months	10.59 (4.79)	9.63 (5.11)	.599
	18 months	9.47 (6.19)	7.23 (4.54)	.268

OA_RI	Baseline	0.79 (0.11)	0.76 (0.11)	.437
	1 month	0.79 (0.07)	0.75 (0.11)	.158
	6 months	0.76 (0.11 )	0.76 (0.09)	.986
	12 months	0.72 (0.12)	0.82 (0.17)	.046*
	18 months	0.76 (0.10)	0.87 (0.28)	.189

CRA_PSV (cm/s)	Baseline	15.09 (3.78)	17.91 (7.80)	.218
	1 month	17.78 (4.43)	18.59 (7.34)	.716
	6 months	19.08 (7.59)	17.67 (5.95)	.575
	12 months	28.88 (13.40)	22.71 (12.82)	.208
	18 months	18.69 (8.79)	17.46 (5.24)	.645

CRA_EDV (cm/s)	Baseline	4.56 (1.81)	6.33 (2.48)	.034*
	1 month	6.49 (2.22)	5.41 (3.19)	.291
	6 months	6.0 (2.49)	6.16 (2.64)	.868
	12 months	7.56 (3.67)	10.31 (7.34)	.204
	18 months	5.66 (2.80)	6.85 (3.24)	.289

CRA_RI	Baseline	0.80 (0.26)	0.81 (0.25)	.915
	1 months	0.68 (0.08)*	0.80 (0.16)*	.011*
	6 months	0.65 (0.082)	0.72 (0.19)	.192
	12 months	0.74 (0.19)	0.85 (0.19)	.000*
	18 months	0.67 (0.09)	0.93 (0.23)	.000*

SPCA_PSV (cm/s)	Baseline	15.55 (4.70)	14.50 (6.59)	.606
	1 month	15.95 (5.91)	13.38 (3.10)	.147
	6 months	20.03 (6.42)	17.92 (3.68)	.280
	12 months	21.01 (10.40)	19.81 (7.04)	.715
	18 months	13.69 (5.45)	11.03 (2.83)	.104

SPCA_EDV (cm/s)	Baseline	4.42 (2.29)	14.50 (6.59)	.973
	1 month	4.69 (2.28)	3.31 (2.11)	.095
	6 months	6.10 (2.16)	5.47 (2.22)	.442
	12 months	6.04 (2.67)*	3.43 (2.26)*	.007*
	18 months	4.39 (1.85)	3.87 (1.17)	.366

SPCA_RI	Baseline	0.71 (0.06)	0.79 (0.28)	.232
	1 month	0.75 (0.08)	0.79 (0.10)	.229
	6 months	0.69 (0.06)	0.69 (0.11)	.969
	12 months	0.70 (0.07)*	0.90 (0.27)*	.011*
	18 months	0.69 (0.11)*	0.85 (0.30)*	.015*

*P < .05 statistically significant.

DTFC: dorzolamide/timolol fixed combination; LTFC: latanoprost/timolol fixed combination; OA: ophthalmic artery; CRA: central retinal artery; SPCA: short posterior ciliary artery, PSV: peak systolic velocity; EDV: end diastolic velocity; RI: resistive index.

**Table 3:** Pulsatile ocular blood flow parameters.

Characteristics		DTFC	LTFC	*P* value
IOP average (mmHg)	baseline	19.58 (3.68)	20.96 (3.78)	.320
	1 month	17.12 (3.25)	18.01 (2.83)	.429
	6 months	17.67 (3.73)	17.71 (3.17)	.975
	12 months	17.87 (3.59)	16.48 (2.56)	.231
	18 months	16.10 (2.78)	15.23 (4.61)	.539

Pulse amplitude	Baseline	4.17 (1.50)	4.73 (1.58)	.335
	1 month	3.91 (0.88)	3.95 (1.18)	.917
	6 months	4.93 (1.88)	4.12 (1.47)	.201
	12 months	4.75 (1.40)	4.67 (1.74)	.891
	18 months	4.73 (2.78)	4.51 (1.42)	.675

Pulse volume (*μ*L)	Baseline	7.19 (2.36)	7.81 (2.68)	.507
	1 month	7.99 (2.27)	7.60 (2.40)	.648
	6 months	8.91 (2.23)	7.07 (3.26)	.417
	12 months	9.25 (1.95)*	6.93 (3.20)*	.025*
	18 months	9.29 (2.39)*	7.82 (1.55)	.054*

POBF Baseline (*μ*L/s)	Baseline	16.81 (4.53)	17.57 (6.13)	.702
	1 month	19.12 (4.45)	18.52 (5.48)	.754
	6 months	19.43 (4.54)	18.63 (6.21)	.69
	12 months	20.87 (4.45)	18.43 (6.51)	.242
	18 months	21.33 (2.74)	19.75 (5.61)	.336

*P < .05 statistically significant.

DTFC: dorzolamide/timolol fixed combination; LTFC: latanoprost/timolol fixed combination; IOP: intraocular pressure; POBF: pulsatile ocular blood flow.

**Table 4:** Comparison of means between progressing and stable glaucoma patients at 18 months visit.

Parameter at 18 month	Mean in stable glaucoma patients (St. deviation)	Mean in progressing glaucoma patients (St. deviation)	*P* value (*t*-test)
IOP	15.32 (2.46)	16.73 (3.04)	.171
IOP/POBF	14.73 (3.5)	16.88 (3.89)	.123

MD (dB)	−1.06 (2.30)	−2.01 (2.13)	.257
PSD (dB)	2.05 (2.53)	2.90 (2.41)	.360

TSNIT (*μ*)	53.59 (5.28)	50.96 (7.10)	.254
NFI	23.82 (2.36)	27.69 (3.29)	.0008*

SBP (mmHg)	151.50 (14.04)	147.73 (20.66)	.55
DBP (mmHg)	88.44 (6.42)	83.53 (8.23)	.077
OPP (mmHg)	57.19 (4.73)	51.84 (7.00)	.019*
DPP (mmHg)	73.06 (6.57)	64.85 (8.82)	.007*

OA_PSV	32.26 (3.15)	29.82 (3.28)	.048
OA_EDV	9.19 (4.98)	7.25 (2.01)	.197
OA_RI	0.74 (0.07)	0.90 (0.07)	<.0001*

CRA_PSV	19.96 (7.36)	15.61 (6.28)	.099
CRA_EDV	6.66 (3.19)	5.73 (2.85)	.415
CRA_RI	0.79 (0.08)	0.815 (0.06)	.35

SPCA_PSV	13.08 (5.11)	11.42 (3.43)	.321
SPCA_EDV	4.73 (1.71)	3.34 (0.83)	.011*
SPCA_RI	0.77 (0.20)	0.76 (0.20)	.893

AMPLITUDE	4.62 (1.48)	4.62 (1.44)	.985
PULSE VOLUME	9.71 (2.01)	7.03 (1.00)	.0001*
POBF	21.25 (4.22)	19.60 (4.60)	.316

DTFC: dorzolamide/timolol fixed combination; LTFC: latanoprost/timolol fixed combination; MD: mean deviation; PSD: pattern standard deviation; TSNIT: temporal, superior, nasal, inferior, temporal average; NFI: nerve fiber index. POBF: pulsatile ocular blood flow.

## References

[1] Drange S, Anderson DR, Schulzer M (2001). Risk factors for progression of visual field abnormalities in normal-tension glaucoma. *American Journal of Ophthalmology*.

[2] Heijl A, Leske MC, Bengtsson B, Hyman L, Bengtsson B, Hussein M (2002). Reduction of intraocular pressure and glaucoma progression: results from the Early Manifest Glaucoma Trial. *Archives of Ophthalmology*.

[3] Leske MC, Heijl A, Hyman L, Bengtsson B, Dong L, Yang Z (2007). Predictors of long-term progression in the early manifest glaucoma trial. *Ophthalmology*.

[4] Flammer J (1994). The vascular concept of glaucoma. *Survey of Ophthalmology*.

[5] Hayreh SS (1994). Progress in the understanding of the vascular etiology of glaucoma. *Current Opinion in Ophthalmology*.

[6] European Glaucoma Society (2008). *Terminology and Guidelines for Glaucoma*.

[7] Osborne NN, Ugarte M, Chao M (1999). Neuroprotection in relation to retinal ischemia and relevance to glaucoma. *Survey of Ophthalmology*.

[8] Siesky B, Harris A, Kheradiya N, Rospigliosi C, McCranor L, Ehrich R (2007). The clinical significance of vascular factors in glaucoma. *Journal of Current Glaucoma Practice*.

[9] Costa VP, Harris A, Stefánsson E (2003). The effects of antiglaucoma and systemic medications on ocular blood flow. *Progress in Retinal and Eye Research*.

[10] Harris A, Arend O, Arend S, Martin B (1996). Effects of topical dorzolamide on retinal and retrobulbar hemodynamics. *Acta Ophthalmologica Scandinavica*.

[11] Martinez A, Gonzalez F, Capeans C, Perez R, Sanchez-Salorio M (1999). Dorzolamide effect on ocular blood flow. *Investigative Ophthalmology & Visual Science*.

[12] Harris A, Arend O, Kagemann L, Garrett M, Chung HS, Martin B (1999). Dorzolamide, visual function and ocular hemodynamics in normal-tension glaucoma. *Journal of Ocular Pharmacology and Therapeutics*.

[13] Harris A, Arend O, Chung HS, Kagemann L, Cantor L, Martin B (2000). A comparative study of betaxolol and dorzolamide effect on ocular circulation in normal-tension glaucoma patients. *Ophthalmology*.

[14] Harris A, Jonescu-Cuypers CP, Kagemann L (2001). Effect of dorzolamide timolol combination versus timolol 0.5% on ocular bloodflow in patients with primary open-angle glaucoma. *American Journal of Ophthalmology*.

[15] Avunduk AM, Sari A, Akyol N (2001). The one-month effects of topical betaxolol, dorzolamide and apraclonidine on ocular blood flow velocities in patients with newly diagnosed primary open-angle glaucoma. *Ophthalmologica*.

[16] Bernd AS, Pillunat LE, Böhm AG, Schmidt KG, Richard G (2001). Okuläre hämodynamik und gesichtsfeld beim glaukom unter dorzolamid-therapie. *Ophthalmologe*.

[17] Pillunat LE, Böhm AG, Köller AU, Schmidt KG, Klemm M, Richard G (1999). Effect of topical dorzolamide on optic nerve head blood flow. *Graefe’s Archive for Clinical and Experimental Ophthalmology*.

[18] Bergstrand IC, Heijl A, Harris A (2002). Dorzolamide and ocular blood flow in previously untreated glaucoma patients: a controlled double-masked study. *Acta Ophthalmologica Scandinavica*.

[19] Siesky B, Harris A, Brizendine E (2009). Literature review and meta-analysis of topical carbonic anhydrase inhibitors and ocular blood flow. *Survey of Ophthalmology*.

[20] Medeiros FA, Alencar LM, Zangwill LM (2009). Detection of progressive retinal nerve fiber layer loss in glaucoma using scanning laser polarimetry with variable corneal compensation. *Investigative Ophthalmology & Visual Science*.

[21] Alencar LM, Zangwill LM, Weinreb RN (2010). A comparison of rates of change in neuroretinal rim area and retinal nerve fiber layer thickness in progressive glaucoma. *Investigative Ophthalmology & Visual Science*.

[22] Januleviciene I, Ehrlich R, Siesky B, Nedzelskiené I, Harris A (2009). Visual function, optic nerve structure, and ocular blood flow parameters after 1 year of glaucoma treatment ith fixed combinations. *European Journal of Ophthalmology*.

[23] Williamson TH, Harris A (1996). Color Doppler ultrasound imaging of the eye and orbit. *Survey of Ophthalmology*.

[24] Pourcelot L (1974). Applications of cliniques de l’examinen Doppler transcutane. *INSERM*.

[25] Pourcelot L (1975). Indications of Doppler ultrasonography in the study of peripheral vessels. *Revue du Praticien*.

[26] Ciula TA, Regillo CD, Harris A (2003). *Retina and Optic Nerve Imaging*.

[27] Siesky B, Harris A, Sines D (2006). A comparative analysis of the effects of the fixed combination of timolol and dorzolamide versus latanoprost plus timolol on ocular hemodynamics and visual function in patients with primary open-angle glaucoma. *Journal of Ocular Pharmacology and Therapeutics*.

[28] Grunwald JE, Piltz J, Hariprasad SM, DuPont J (1998). Optic nerve and choroidal circulation in glaucoma. *Investigative Ophthalmology & Visual Science*.

[29] Michelson G, Langhans MJ, Harazny J, Dichtl A (1998). Visual field defect and perfusion of the juxtapapillary retina and the neuroretinal rim area in primary open-angle glaucoma. *Graefe’s Archive for Clinical and Experimental Ophthalmology*.

[30] Hayreh SS, Revie IH, Edwards J (1970). Vasogenic origin of visual field defects and optic nerve changes in glaucoma. *British Journal of Ophthalmology*.

[31] Yaoeda K, Shirakashi M, Fukushima A (2003). Relationship between optic nerve head microcirculation and visual field loss in glaucoma. *Acta Ophthalmologica Scandinavica*.

[32] Nicolela MT, Drance SM, Rankin SJA, Buckley AR, Walman BE (1996). Color Doppler imaging in patients with asymmetric glaucoma and unilateral visual field loss. *American Journal of Ophthalmology*.

[33] Yamazaki Y, Drance SM (1997). The relationship between progression of visual field defects and retrobulbar circulation in patients with glaucoma. *American Journal of Ophthalmology*.

[34] Sommer A, Katz J, Quigley HA (1991). Clinically detectable nerve fiber atrophy precedes the onset of glaucomatous field loss. *Archives of Ophthalmology*.

[35] Hafez AS, Bizzarro RLG, Lesk MR (2003). Evaluation of optic nerve head and peripapillary retinal blood flow in glaucoma patients, ocular hypertensives, and normal subjects. *American Journal of Ophthalmology*.

[36] Yamazaki Y, Hayamizu F (1995). Comparison of flow velocity of ophthalmic artery between primary open angle glaucoma and normal tension glaucoma. *British Journal of Ophthalmology*.

[37] Nicolela MT, Walman BE, Buckley AR, Drance SM (1996). Ocular hypertension and primary open-angle glaucoma: a comparative study of their retrobulbar blood flow velocity. *Journal of Glaucoma*.

[38] Rankin SJA, Walman BE, Buckley AR, Drance SM (1995). Color Doppler imaging and spectral analysis of the optic nerve vasculature in glaucoma. *American Journal of Ophthalmology*.

[39] Rojanapongpun P, Drance SM, Morrison BJ (1993). Ophthalmic artery flow velocity in glaucomatous and normal subjects. *British Journal of Ophthalmology*.

[40] Kaiser HJ, Schoetzau A, Stumpfig D, Flammer J (1997). Blood-flow velocities of the extraocular vessels in patients with high-tension and normal-tension primary open-angle glaucoma. *American Journal of Ophthalmology*.

[41] Satilmis M, Orgül S, Doubler B, Flammer J (2003). Rate of progression of glaucoma correlates with retrobulbar circulation and intraocular pressure. *American Journal of Ophthalmology*.

[42] Zeitz O, Galambos P, Wagenfeld L (2006). Glaucoma progression is associated with decreased blood flow velocities in the short posterior ciliary artery. *British Journal of Ophthalmology*.

[43] Nielsen PJ, Nyborg NCB (1996). Effects of prostaglandins in bovine isolated aqueous veins. *Investigative Ophthalmology & Visual Science*.

[44] Remky A, Plange N, Klok J, Arend O (2004). Retinal arterial diameters in patients with glaucoma. *Spektrum der Augenheilkunde*.

[45] Klein BEK, Klein R, Knudtson MD (2005). Intraocular pressure and systemic blood pressure: longitudinal perspective: the Beaver Dam Eye Study. *British Journal of Ophthalmology*.

[46] Doshi V, Ying-Lai M, Azen SP, Varma R (2008). Sociodemographic, family history, and lifestyle risk factors for open-angle glaucoma and ocular hypertension. the Los Angeles Latino eye study. *Ophthalmology*.

[47] Topouzis F, Coleman AL, Harris A (2006). Association of blood pressure status with the optic disk structure in non-glaucoma subjects: the Thessaloniki Eye Study. *American Journal of Ophthalmology*.

[48] Choi J, Kim KH, Jeong J, Cho HS, Lee CH, Kook MS (2007). Circadian fluctuation of mean ocular perfusion pressure is a consistent risk factor for normal-tension glaucoma. *Investigative Ophthalmology & Visual Science*.

